# Impacts of Interleukin-10 Promoter Genotypes on Prostate Cancer

**DOI:** 10.3390/life14081035

**Published:** 2024-08-20

**Authors:** Yu-Ting Chin, Chung-Lin Tsai, Hung-Huan Ma, Da-Chuan Cheng, Chia-Wen Tsai, Yun-Chi Wang, Hou-Yu Shih, Shu-Yu Chang, Jian Gu, Wen-Shin Chang, Da-Tian Bau

**Affiliations:** 1 Graduate Institute of Biomedical Sciences, China Medical University, Taichung 404333, Taiwan; 2 Terry Fox Cancer Research Laboratory, Department of Medical Research, China Medical University Hospital, Taichung 404327, Taiwan; 3 Division of Cardiac and Vascular Surgery, Cardiovascular Center, Taichung Veterans General Hospital, Taichung 407219, Taiwan; 4 Division of Nephrology, Department of Internal Medicine, Taichung Tzu Chi Hospital, Taichung 427003, Taiwan; 5 Department of Biomedical Imaging and Radiological Science, China Medical University, Taichung 404333, Taiwan; 6 Department of Epidemiology, The University of Texas MD Anderson Cancer Center, Houston, TX 77030, USA; jiangu@mdanderson.org; 7 Department of Nephrology, Chang-Hua Hospital, Ministry of Health and Welfare, Changhua 51341, Taiwan; 8 Department of Bioinformatics and Medical Engineering, Asia University, Taichung 413305, Taiwan

**Keywords:** genotype, interleukin-10, SNP, prostate cancer

## Abstract

Prostate cancer (PCa) is a multifactorial disease influenced by genetic, environmental, and immunological factors. Genetic polymorphisms in the *interleukin-10* (*IL-10*) gene have been implicated in PCa susceptibility, development, and progression. This study aims to assess the contributions of three *IL-10* promoter single nucleotide polymorphisms (SNPs), A-1082G (rs1800896), T-819C (rs3021097), and A-592C (rs1800872), to the risk of PCa in Taiwan. The three *IL-10* genotypes were determined using PCR-RFLP methodology and were evaluated for their contributions to PCa risk among 218 PCa patients and 436 non-PCa controls. None of the three *IL-10* SNPs were significantly associated with the risks of PCa (*p* all > 0.05) in the overall analyses. However, the GG at rs1800896 combined with smoking behavior was found to significantly increase the risk of PCa by 3.90-fold (95% confidence interval [95% CI] = 1.28–11.89, *p* = 0.0231). In addition, the rs1800896 AG and GGs were found to be correlated with the late stages of PCa (odds ratio [OR] = 1.90 and 6.42, 95% CI = 1.05–3.45 and 2.30–17.89, *p* = 0.0452 and 0.0003, respectively). The *IL-10* promoter SNP, A-1082G (rs1800896), might be a risk factor for PCa development among smokers and those at late stages of the disease. These findings should be validated in larger and more diverse populations.

## 1. Introduction

Prostate cancer (PCa) is the second most common cancer among men worldwide and is a leading cause of cancer-related mortality. The incidence of PCa is increasing in developed countries. It is estimated that the number of PCa cases in men in the United States will reach 299,010, accounting for 29% of all cancers in 2024 [1]. Globally, there were approximately 1,466,680 new PCa cases in 2022 [2]. PCa is difficult to diagnose early. Studies have found that PCa may be related to family history, race, occupation, cadmium exposure, vasectomy, obesity, alcohol consumption, and other factors, with genetic factors possibly being the most important, though largely unrevealed [3,4,5,6,7]. 

The vast majority of PCas are adenocarcinomas originating from glandular cells that line the prostate gland and its tubes. Prostate carcinogenesis is a multi-stage process that begins with the development of low-grade prostatic intraepithelial neoplasia (PIN), followed by localized adenocarcinoma, and ultimately advances to metastatic disease. The pathophysiology of PCa involves complex interactions between genetic, epigenetic, hormonal, and environmental factors. Numerous genetic and epigenetic alterations have been documented in prostate tumors [8]. The most common PCa genetic alterations are translocations involving androgen-regulated promoters and the ETS family of transcription factors, such as ERG and the ETV genes [9], among which the TMPRSS2:ERG fusion occurs in approximately 50% of localized PCa tumors [10,11]. Other frequent somatic genetic alterations include MYC amplification and mutations in pathways of androgen receptor (AR), PI3K–PTEN, TGF-β/SMAD4, DNA repair, and epigenetic regulators and chromatin remodelers [8]. Because a normal prostate needs androgen and its receptor for homeostasis, targeting the AR pathway via androgen deprivation therapy (ADT) is the standard of care for PCa. Resistance to ADT results in castration-resistant PCa (CRPC) and metastatic CRPC (mCRPC), which accounts for most of the PCa death.

We have previously reported a genome-wide association study of PCa [12] together with several candidate genes [13,14,15,16,17] among Taiwanese; however, the genetic contributions to PCa susceptibility and prognosis in Taiwanese individuals are far from satisfying and need further investigation. Interleukin-10 (IL-10) is a pivotal cytokine involved in immune regulation, exerting dual roles in both promoting and inhibiting immune responses. Its impact on tumorigenesis is paradoxical, as it can both facilitate and suppress tumor development [18]. IL-10 can inhibit tumorigenesis by enhancing CD8+ T cell activity and suppressing pro-inflammatory cytokines such as IL-6 and IL-23 [19]. Conversely, IL-10 can hinder antigen presentation and suppress interferon gamma (IFN-γ), thereby potentially dampening anti-tumor immunity [19]. In the context of PCa, elevated IL-10 levels in both tissue and serum have long been recognized as prognostic indicators of PCa aggressiveness and progression [20,21,22,23,24,25]. Over-expression of IL-10 has been associated with PCa development in several studies among different populations [20,21,22,23,24,25]; however, the genotype-phenotype correlation is not well known. The *IL-10* gene comprises five exons and is located on chromosome 1q31-32 [26]. Three commonly studied SNPs in the promoter region of the *IL-10* gene, rs1800896, rs3021097 and rs1800872, have been reported to modulate its expression [27,28].

In this study, we examined the associations of three *IL-10* promoter SNPs, rs1800896, rs3021097 and rs1800872, with the risk and aggressiveness of PCa in Taiwanese. In addition, we investigated the combinative effects of *IL-10* genotype with age and smoking behavior on PCa risk and aggressiveness. Finally, we performed a comprehensive literature review of the associations of rs1800896 with PCa risk.

## 2. Materials and Methods

### 2.1. Recruitment of PCa Cases and Healthy Controls

In this study, 218 patients diagnosed with PCa were recruited from the outpatient clinics of the general surgery at China Medical University Hospital, Taichung, Taiwan. The recruitment was facilitated by the hospital’s Tissue Bank. During the same period, 436 healthy volunteers without PCa were recruited as controls. These controls were matched by age and were selected through initial random sampling from the Health Examination Cohort of the same hospital. The details of the inclusion and exclusion criteria have been published previously [12,13]. Briefly, the exclusion criteria for the control group included a history of malignancy, metastasized cancer of known or unknown origin, and any familial or genetic diseases. Additionally, control subjects lacking the specified demographic data were excluded from the study. All participants were Taiwanese citizens and the population in Taiwan is racially homogeneous. The study design and protocols have been approved and supervised by the Institutional Review Board of China Medical University Hospital (CMUH110-REC3-005). Several selected characteristics including age, smoking behaviors, family history, disease stages, and pathologic grades are summarized in Table 1.

### 2.2. IL-10 Genotyping Methodology

**Figure 1:**
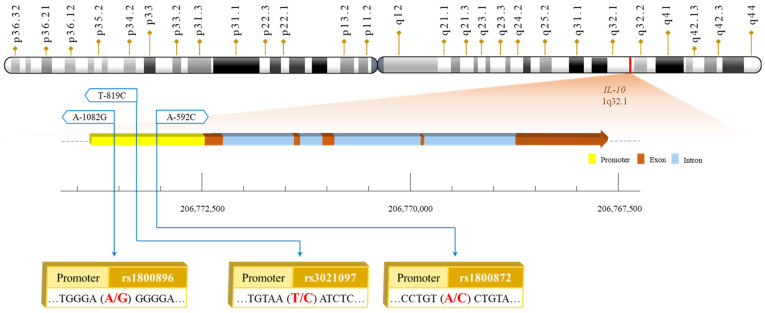
Physical map for *IL-10* rs1800896, rs3021097, and rs1800872 promoter polymorphic sites.

Genomic DNA was extracted from the peripheral blood of each participant as our published routine protocol [29,30]. The genotypes of *IL-10* rs1800896, rs3021097, and rs1800872 were determined using polymerase chain reaction-restriction fragment length polymorphism (PCR-RFLP) methodology, as we have previously described [31]. The PCR primer sequences and respective restriction endonuclease for each DNA product of *IL-10* rs1800896, rs3021097, and rs1800872 SNPs are summarized in Table 2. The locations of the investigated *IL-10* polymorphic sites are summarized in Figure 1.

### 2.3. Selection of the Literature

Published articles were chosen from PubMed via EndNote, updated to 1 July 2024. The exclusion criteria were as follows: (a) articles published in non-SCI journals; (b) articles without a well-defined investigated population; (c) articles investigating small sample populations with insufficient analyzing power (fewer than 150 controls or cases, or fewer than 400 total samples); and (d) articles where the genotypic distribution does not fit the Hardy–Weinberg equilibrium (HWE).

### 2.4. Statistical Analysis

We used Student’s *t*-test to compare the mean age and the Pearson’s Chi-square test to compare the genotype frequency between the PCa case and non-PCa control groups. The association between *IL-10* genotypes and PCa risk was determined using a multivariable logistic regression analysis. The tests were two-sided, and any *p*-value less than 0.05 was considered to be statistically significant.

## 3. Results

### 3.1. Demographics and Lifestyles for the PCa Case and Non-PCa Control Groups

The distributions of demographic characteristics, including age, smoking behaviors, and family history for the 218 PCa patients and 436 non-PCa healthy controls, are compared in Table 1. Additionally, the disease stages and pathologic grades of the PCa cases are also shown in Table 1. The mean age ± standard deviation of the cases and controls were 63.6 ± 6.9 and 63.9 ± 6.6, respectively (*p* = 0.58). About 81% of the cases are ever smokers, compared to 77% for the controls (*p* = 0.27). Among the PCa cases, 7.8% and 1.8% had a family history of any cancer in their first- and second-degree relatives, respectively; 71.1% and 28.9% were in the early and late stages, respectively; and 12.9%, 40.8%, and 46.3% of the PCa cases were of well-, moderately, and poorly differentiated grades, respectively (Table 1).

### 3.2. Contributions of IL-10 Genotypes to PCa Risk

Table 3 reveals the distributions of *IL-10* genotypes among the PCa patients and non-PCa control subjects. All the three *IL-10* genotypes fit well with the Hardy–Weinberg equilibrium in the controls (*p* = 0.3428, 0.2680, and 0.6034, respectively). The distributions of the three genotypes were not significantly different between the cases and controls (*P*_trend_ = 0.1755, 0.8296, and 0.6547, respectively) (Table 3). There were suggestive associations between the rs1800896 genotypes and PCa risk: the homozygous variant GG and heterozygous variant AG genotype were associated with elevated risks of PCa (adjusted OR = 1.18 and 1.98, 95% CI = 0.79–1.68 and 0.85–3.75, *p*-value = 0.3933 and 0.1451, respectively) (Table 3).

### 3.3. Contributions of IL-10 Alleles to PCa Risk

The allelic frequencies of the three *IL-10* SNPs among the PCa patients and non-PCa healthy controls were also examined (Table 4). In line with the findings in Table 3, the rs3021097 and rs1800872 allelic frequencies was not significantly different between the PCa case and non-PCa control groups (*p* = 0.6106 and 0.3885, respectively) (Table 4). However, there was a suggestive association between the variant G allele of rs1800896 and an increased risk of PCa (OR = 1.33, 95% CI = 0.97–1.83, *p* = 0.0906).

### 3.4. Association of IL-10 rs1800896 Genotypes with PCa Risk Stratified by Clinicopathologic Characteristics

We further analyzed the association of rs1800896 genotypes with PCa risk stratified by clinicopathologic characteristics including age, smoking behavior, and disease stage (Table 5). Interestingly, a significant association was observed in smokers, but not in non-smokers. The ORs (95% CI) for the heterozygous variant genotype (AG) and homozygous variant genotype (GG) were 1.46 (0.96–2.24) and 3.90 (1.28–11.89), *p* = 0.0983 and 0.0231, respectively. In addition, the AG and GGs were associated with a significantly increased risk of PCa in late-stage patients (adjusted OR = 1.92, 95% CI = 1.09–3.68, *p* = 0.0452; and adjusted OR = 5.81, 95% CI = 2.54–14.77, *p* = 0.0003, respectively), but not in early-stage patients.

## 4. Discussion

In recent years, new advances in molecular and genetic studies have demonstrated a causal relationship between chronic infection, chronic inflammation, and PCa [32,33]. Chronic inflammation has been hypothesized to be a cause of PCa, contributing to carcinogenesis during both disease initiation and subsequent progression [34,35]. Over-expression of IL-10 has been associated with elevated PCa risk [20,21,22,23,24,25]. Elevated IL-10 levels have been detected in the serum of PCa patients, correlating with poor prognosis and higher Gleason scores [22,23,24]. In addition, the expression of IL-10 is significantly higher in PCa tumor tissues than in normal prostates and benign prostate lesions and correlates with a high grade and stage of prostate carcinoma [21,36]. IL-10 may be produced by either tumor cells themselves or by immune cells [21,37,38,39,40,41]. IL-10 is predominantly expressed in monocytes and Th2 lymphocytes [42,43]. IL-10 promotes tumor growth in PCa by suppressing the antitumor immune response through its effects on immune cells [44] and by directly acting on the PCa cells [45]. Its suppression of the antitumor immune response includes the suppression of myeloid and T effector cell function [46,47]. IL-10 can upregulate the expression of PDL1 on myeloid cells [48], whereas PDL1 binds to its receptor PD1 on T cells, inhibiting T cell function and hence its antitumor immune response [49]. IL-10 can also directly act on prostate cancer cells. For example, it can induce neuroendocrine differentiation, inhibit AR activity, and upregulate PDL1 in PCa cells, thereby promoting PCa progression [50].

In this study, the association of three commonly studied *IL-10* promoter SNPs and PCa risk was examined among a Taiwanese population containing 218 PCa cases and 436 non-PCa healthy controls. None of the SNPs were significantly associated with the risks of PCa, although a suggestive association between rs1800896 and the risk of PCa was observed (Table 3 and Table 4). In the literature, the rs1800896 AA genotype has been associated with a higher risk of developing PCa in Croatian [51] and Indian populations [52]. However, these two articles had limitations: one had a small sample size (control:case = 120:120), and the distribution of genotypes in the controls in the other did not fit HWE. On the contrary, it has been reported that the rs1800896 AA genotype was associated with decreased risks of PCa in USA [53], UK [54], and Indian populations [55]. Several other studies of populations of European ancestry [56,57,58,59,60,61,62] and a study of Chinese populations [63] did not find significant associations (Table 6). These heterogeneous results may be due to small sample sizes, diverse populations, differing genetic architecture, and a range of environmental exposures.

Regarding the biochemical mechanisms underlying our observed association, the literature has consistently demonstrated that individuals with the *IL-10* rs1800896 (A-1082G) variant AG and GGs have elevated serum levels of IL-10 compared to those with the wild-type AA genotype [64,65,66,67]. Moreover, the rs1800896 SNP is located within the transcriptional factor binding sites and the variant G-allele has higher transcriptional activity than the A-allele in various cell lines [68,69,70,71]. These data strongly support our observations that the variant genotypes AG and GG of this SNP correlate with late stages of PCa and significantly increase the risk of PCa in smokers, because IL-10 is a pro-tumorigenic cytokine in PCa and the variant genotypes increase IL-10 level, thus promoting PCa development and progression.

To investigate how these SNPs modulate IL-10 expression, Reuss et al. [68] performed a thorough study characterizing the three *IL-10* promoter SNPs on transcriptional activity and their bindings to transcriptional factors using luciferase reporter gene and electrophoretic mobility shift assays (EMSA). They found that the rs1800896 (A-1082G) lies within an ETS-consensus binding site and that the A-allele specifically binds to an ETS-family transcriptional factor SPI1/PU.1, whereas the G-allele does not. They reasoned that the differential SPI1 binding accounts for the different transcriptional activity and that SPI1 binding to the A-allele negatively regulates IL-10 expression. The negative transcriptional regulation by SPI1 binding has also been reported for other genes [72,73]. In addition, several studies have shown that another transcription factor, Sp1, only bound to the G allele in response to inflammatory stimulation, resulting in a much larger increase in IL-10 mRNA and protein levels in B-cell lines with the GG than the AA genotype [69,70,71]. It appears that both positive and negative transcriptional regulation contribute to the higher transcriptional activity of the G-alleles of rs1800896. 

The associations of the other two SNPs, rs3021097 (T-819C) and rs1800872 (A-592C), with IL-10 expression were inconsistent in the literature [74,75]. They are not located within transcription factor binding sites and do not bind to transcriptional factors in EMSA experiments [68]. It is thus not surprising that we did not observe any significant associations of these two SNPs with PCa risk. 

There have been lots of epidemiological studies investigating the associations between *IL-10* promoter SNPs, which may alter the function of this cytokine itself and its related cellular behavior and lead to the development of human disorders. The rs1800896 has been reported to be associated with oral cancer [76,77], nasopharyngeal carcinoma [78], papillary thyroid cancer [79,80], gastric cancer [81,82,83], lung cancer [84], breast cancer [85], renal cell carcinoma [86,87], cervical cancer [88,89], lymphoma [90,91], and leukemia [92,93]. The rs3021097 was reported to be associated with gastric cancer [84,94,95], renal cell carcinoma [96], lung cancer [97], cervical cancer [98], bladder cancer [99], and leukemia [28,100]. The rs1800872 was associated with oral cancer [101], esophageal cancer [102], gastric cancer [103], lung cancer [84], breast cancer [104], cervical cancer [105,106], lymphoma [107], and leukemia [100]. Future larger studies would be necessary to clarify the roles of these SNPs in cancer predisposition due to their modest effect size. In addition, collecting comprehensive clinical and pathological data for all cases, along with full follow-up and periodic assessment of therapeutic responses, is crucial for precise genomics. Recruiting appropriately matched control subjects in terms of age, gender, and lifestyle behaviors is extremely important. Furthermore, a proper combination of multiple SNPs in a single candidate gene, such as *IL-10* rs1800896, rs3021097, and rs1800872 in this study, can provide informative coverage of the entire gene.

IL-10 is a major suppressor of immune response and plays important roles in a variety of human diseases, thus becoming an attractive therapeutic target [108,109]. In an IL-10 transgenic mouse model, high expression of IL-10 failed to control an immunogenic lung tumor; however, administering an anti-IL-10 antibody significantly inhibited tumor growth [110]. Likewise, IL-10 knock-out mice are resistant to UV-induced skin carcinogenesis, correlating with a potent Th1 response in these mice [111]. Ruffell et al. [112] showed that blocking IL-10 signaling using an IL-10 receptor (IL-10R) monoclonal antibody enhanced paclitaxel and carboplatin efficacy in a breast cancer model. Blocking IL-10 could also suppress the metastatic behaviors of colorectal cancer cells [113] and promote gastrointestinal cancer cell death [114]. These data support the concept that IL-10 suppresses the anti-tumoral immune response, and blocking IL-10 can strengthen the anti-tumoral immune response and inhibit tumor growth. However, it should be cautioned that IL-10 plays a dual role in human carcinogenesis depending on specific cell types and contexts. It can inhibit certain cancers, and a PEG-conjugated human IL-10 has been on clinical trial for pancreatic cancer [109,115]. Future studies should test the clinical potential of targeting IL-10 and its downstream molecules in the context of PCa and investigate the pleiotropic effects of IL-10 in different cancers.

The well-established risk factors for PCa include age, ethnicity, family history, genetic predisposition, diet, and environmental factors [116,117,118,119,120,121]. We performed stratified analyses on age and did not find significant differences in the associations of these SNPs with PCa risks in young and old aged groups. Smoking is inversely associated with overall PCa incidence, but may lead to more aggressive PCa tumors and worse prognosis of PCa patients [122,123,124]. Our stratified analyses demonstrated that the variant genotypes at rs1800896 increases the likelihood of PCa among smokers (Table 6), and are associated with increased risks of late-stage PCa (Table 5). In clinical practice, determining *IL-10* genotypes alongside smoking history may aid in developing precise personalized screening and prevention strategies against aggressive PCa. The molecular mechanisms underlying the associations of the *IL-10* rs1800896 variant genotypes with PCa risk in smokers and with aggressive PCa remains unclear and warrants future investigation.

## 5. Conclusions

In conclusion, this is the first report of three *IL-10* promoter SNPs in relation to PCa susceptibility in Taiwanese. We found significant associations of the *IL-10* rs1800896 variant genotypes with PCa risk in smokers and with aggressive PCa. Further studies are necessary to validate the *IL-10* genetic variants as PCa predisposition markers in diverse populations and investigate the molecular mechanisms underlying the observed associations.

## Figures and Tables

**Table 1:** Demographics of prostate cancer cases and control subjects.

Characteristics	Controls (n = 436)			Cases (n = 218)			*p*-Value
	n	%	Mean ± SD	n	%	Mean ± SD	
Age (years)			63.9 ± 6.6			63.6 ± 6.9	0.58 ^a^
<55	275	63.1%		142	65.1%		0.67 ^b^
>55	161	36.9%		76	34.9%		
Smoking behavior							
Ever smoker	336	77.0%		177	81.2%		0.27 ^b^
Non-smoker	100	23.0%		41	18.8%		
Family history							
First degree (father, brother, and/or son)				17	7.8%		
Second degree				4	1.8%		
No history				197	90.4%		
Disease stage							
Early stage				155	71.1%		
Late stage				63	28.9%		
Pathologic grade							
Well-differentiated				28	12.9%		
Moderately differentiated				89	40.8%		
Poorly differentiated				101	46.3%		

SD: standard deviation; ^a^ based on unpaired Student’s *t*-test; ^b^ based on Chi-square test.

**Table 2:** The primer sequences, polymerase chain reaction, and restriction fragment length polymorphism methodology for identifying *interleukin-10* rs1800896, rs3021097, and rs1800872 genotypes.

Polymorphic Cites (rs Numbers)	Primer Sequences	Endonuclease	Polymorphic Pattern	Digested Adduct Size (bp)
A-1082G (rs1800896)	F: 5′-CTCGCTGCAACCCAACTGGC-3′ R: 5′-TCTTACCTATCCCTACTTCC-3′	*Mnl* I	A G	139 106 + 33
T-819C (rs3021097)	F: 5′-TCATTCTATGTGCTGGAGAT-3′ R: 5′-TGGGGGAAGTGGGTAAGAGT-3′	*Mae* III	T C	209 125 + 84
A-592C (rs1800872)	F: 5′-GGTGAGCACTACCTGACTAG-3′ R: 5′-CCTAGGTCACAGTGACGTGG-3′	*Rsa* I	C A	412 236 + 176

F and R stands for forward and reverse primers, respectively.

**Table 3:** Distributions of *interleukin-10* genotypic frequencies among prostate cancer patients and healthy controls.

	Cases (%)	Controls (%)	OR (95% CI)	Adjusted OR (95% CI) ^a^	*p*-Value ^b^
rs1800896					
AA	154 (70.6)	330 (75.7)	1.00 (reference)	1.00 (reference)	
AG	54 (24.8)	96 (22.0)	1.20 (0.82–1.77)	1.18 (0.79–1.68)	0.3933
GG	10 (4.6)	10 (2.3)	2.14 (0.87–5.26)	1.98 (0.85–3.75)	0.1451
AG+GG	64 (29.4)	106 (24.3)	1.29 (0.90–1.86)	1.27 (0.89–1.84)	0.1962
*P*_trend_					0.1755
*P*_HWE_					0.3428
rs3021097					
TT	116 (53.2)	243 (55.7)	1.00 (reference)	1.00 (reference)	
TC	84 (38.5)	159 (36.5)	1.10 (0.78–1.56)	1.07 (0.73–1.38)	0.6253
CC	18 (8.3)	34 (7.8)	1.11 (0.60–2.05)	1.09 (0.70–1.48)	0.8627
TC+CC	102 (46.8)	193 (44.3)	1.11 (0.80–1.53)	1.09 (0.74–1.39)	0.5976
*P*_trend_					0.8296
*P*_HWE_					0.2680
rs1800872					
AA	126 (57.8)	267 (61.2)	1.00 (reference)	1.00 (reference)	
AC	78 (35.8)	146 (33.5)	1.13 (0.80–1.60)	1.09 (0.78–1.57)	0.5406
CC	14 (6.4)	23 (5.3)	1.29 (0.64–2.59)	1.27 (0.61–2.46)	0.5938
AC+CC	92 (42.2)	169 (38.8)	1.15 (0.83–1.61)	1.11 (0.81–1.58)	0.4459
*P*_trend_					0.6547
*P*_HWE_					0.6034

OR: odds ratio; CI: confidence interval; ^a^ data based on Chi-square test with Yates’ correction; ^b^ adjusted for age; *P*_trend_: *p*-value based on trend analysis; *P*_HWE_: *p*-value based on Hardy–Weinberg Equilibrium.

**Table 4:** Distributions of allelic frequencies for *interleukin-10* polymorphisms in prostate cancer patients and control groups.

Polymorphic Site Allele	Cases (%) N = 436	Controls (%) N = 872	OR (95% CI)	*p*-Value ^a^
rs1800896				
A	362 (83.0)	756 (86.7)	1.00 (reference)	
G	74 (17.0)	116 (13.3)	1.33 (0.97–1.83)	0.0906
rs3021097				
T	316 (72.5)	645 (74.0)	1.00 (reference)	
C	120 (27.5)	227 (26.0)	1.08 (0.83–1.40)	0.6106
rs1800872				
A	330 (75.7)	680 (78.0)	1.00 (reference)	
C	106 (24.3)	192 (22.0)	1.14 (0.87–1.49)	0.3885

OR, odds ratio; CI, confidence interval. ^a^ *p*-value based on Chi-square test with Yate’s correction.

**Table 5:** Association of *interleukin-10* rs1800896 genotypes with prostate cancer risk stratified by clinicopathologic characteristics.

	Controls (%)	Cases (%)	OR (95% CI)	Adjusted OR (95% CI) ^a^	*p*-Value ^b^
**Age**					
<55 (years)					
AA	211 (76.7)	106 (74.7)	1.00 (reference)	1.00 (reference)	
AG	59 (21.4)	32 (22.5)	1.08 (0.66–1.76)	1.07 (0.63–1.68)	0.8563
GG	5 (1.8)	4 (2.8)	1.59 (0.42–6.05)	1.52 (0.32–5.74)	0.7405
≥55 (years)					
AA	119 (73.9)	48 (63.2)	1.00 (reference)	1.00 (reference)	
AG	37 (23.0)	22 (28.9)	1.47 (0.79–2.75)	1.41 (0.73–2.38)	0.2907
GG	5 (3.1)	6 (7.9)	2.97 (0.87–10.21)	2.58 (0.56–8.74)	0.1430
**Smoking behaviors**					
Non-smokers					
AA	70 (70.0)	34 (82.9)	1.00 (reference)	1.00 (reference)	
AG	25 (25.0)	6 (14.7)	0.49 (0.19–1.32)	0.44 (0.18–1.28)	0.2288
GG	5 (5.0)	1 (2.4)	0.41 (0.05–3.66)	0.39 (0.05–3.58)	0.6624
Smokers					
AA	260 (77.4)	120 (67.8)	1.00 (reference)	1.00 (reference)	
AG	71 (21.1)	48 (27.1)	1.46 (0.96–2.24)	1.38 (0.89–2.08)	0.0983
GG	5 (1.5)	9 (5.1)	**3.90 (1.28–11.89)**	**3.67 (1.25–8.75)**	**0.0231**
**Disease stages**					
Early stage					
AA	330 (75.7)	118 (76.1)	1.00 (reference)	1.00 (reference)	
AG	96 (22.0)	34 (21.9)	0.99 (0.64–1.54)	1.03 (0.71–1.62)	0.9663
GG	10 (2.3)	3 (2.0)	0.84 (0.23–3.10)	0.91 (0.19–3.25)	1.0000
Late stage					
AA	330 (75.7)	36 (57.1)	1.00 (reference)	1.00 (reference)	
AG	96 (22.0)	20 (31.8)	**1.90 (1.05–3.45)**	**1.92 (1.09–3.68)**	**0.0452**
GG	10 (2.3)	7 (11.1)	**6.42 (2.30–17.89)**	**5.81 (2.54–14.77)**	**0.0003**

OR, odds ratio; CI, confidence interval. ^a^ Based on Chi-square test with Yate’s correction (all n ≥ 5) or Fisher’s exact test (any n < 5); ^b^ adjusted for other factors (age, smoking, or stage); *P*_trend_: *p*-value based on trend analysis; *P*_HWE_: *p*-value based on Hardy–Weinberg equilibrium; Bolded, statistically significant.

**Table 6:** Concise summary of the literature for association between *interleukin-10* rs1800896 genotypes and prostate cancer.

Author	Year	Country	Ethnics	SOC	Controls (GG, AG, AA)	Cases (GG, AG, AA)	Association Highlights
Chin	2024	Taiwan	East Asian	HB	10:96:330	10:54:154	No association
Winchester	2017	USA	Caucasian	PB	140:254:134	136:305:179	No association
Dluzniewski	2012	USA	Mixed	PB	104:242:112	100:212:146	A allele is risky
Vancleave	2010	USA	Mixed	PB	288:280:92	75:95:22	No association
Liu	2010	China	East Asian	PB	4:36:222	3:27:240	No association
Wang	2009	USA	Caucasian	PB	56:130:69	83:117:57	A allele is protective
Zabaleta	2008	USA	Caucasian	HB	86:206:102	126:239:110	No association
Faupel-Badger	2008	USA	Caucasian	PB	85:251:173	73:194:115	No association
Michaud	2006	USA	Mixed	PB	290:599:356	383:857:523	No association
Xu	2005	Sweden	Caucasian	PB	306:689:388	187:390:203	No association
McCarron	2002	UK	Caucasian	PB	56:113:78	57:120:46	A allele is protective

SOC: source of controls; PB: population based; HB: hospital based.

## Data Availability

The genotyping results and clinical data supporting the findings of this study are available from the corresponding author upon reasonable requests via email at 013280@tool.caaumed.org.tw.

## References

[1] Siegel R.L., Giaquinto A.N., Jemal A. (2024). Cancer statistics, 2024. CA Cancer J. Clin..

[2] Bray F., Laversanne M., Sung H., Ferlay J., Siegel R.L., Soerjomataram I., Jemal A. (2024). Global cancer statistics 2022: GLOBOCAN estimates of incidence and mortality worldwide for 36 cancers in 185 countries. CA Cancer J. Clin..

[3] Fullwood D., Gunderson J., Snipe B., Villasenor I., Asto-Flores E., Pressey S., Hale R., Odedina F.T. (2023). Prostate Cancer Screening and Strategies, Florida Behavioral Risk Factor Surveillance System, 2012–2020. Prev. Chronic Dis..

[4] Zhao J., Stockwell T., Roemer A., Chikritzhs T. (2016). Is alcohol consumption a risk factor for prostate cancer? A systematic review and meta-analysis. BMC Cancer.

[5] Dombi G.W., Rosbolt J.P., Severson R.K. (2010). Neural network analysis of employment history as a risk factor for prostate cancer. Comput. Biol. Med..

[6] Krstev S., Knutsson A. (2019). Occupational Risk Factors for Prostate Cancer: A Meta-analysis. J. Cancer Prev..

[7] Guess H.A. (1993). Is vasectomy a risk factor for prostate cancer?. Eur. J. Cancer.

[8] Wang G., Zhao D., Spring D.J., DePinho R.A. (2018). Genetics and biology of prostate cancer. Genes Dev..

[9] Sizemore G.M., Pitarresi J.R., Balakrishnan S., Ostrowski M.C. (2017). The ETS family of oncogenic transcription factors in solid tumours. Nat. Rev. Cancer.

[10] Tomlins S.A., Rhodes D.R., Perner S., Dhanasekaran S.M., Mehra R., Sun X.W., Varambally S., Cao X., Tchinda J., Kuefer R. (2005). Recurrent fusion of TMPRSS2 and ETS transcription factor genes in prostate cancer. Science.

[11] Tomlins S.A., Bjartell A., Chinnaiyan A.M., Jenster G., Nam R.K., Rubin M.A., Schalken J.A. (2009). ETS gene fusions in prostate cancer: From discovery to daily clinical practice. Eur. Urol..

[12] Bau D.T., Tsai C.W., Chang W.S., Yang J.S., Liu T.Y., Lu H.F., Wang Y.W., Tsai F.J. (2024). Genetic susceptibility to prostate cancer in Taiwan: A genome-wide association study. Mol. Carcinog..

[13] Liao C.H., Chang W.S., Hsu W.L., Hu P.S., Wu H.C., Hsu S.W., Wang B.R., Yueh T.C., Chen C.H., Hsia T.C. (2023). Association of Matrix Metalloproteinase-7 Genotypes With Prostate Cancer Risk. Anticancer Res..

[14] Li P.H., Liao C.H., Huang W.C., Chang W.S., Wu H.C., Hsu S.W., Chen K.Y., Wang Z.H., Hsia T.C., Bau D.T. (2023). Association of Matrix Metalloproteinase-2 Genotypes With Prostate Cancer Risk. Anticancer Res..

[15] Liao C.H., Wu H.C., Hu P.S., Hsu S.W., Shen T.C., Hsia T.C., Chang W.S., Tsai C.W., Bau D.T. (2018). The Association of Matrix Metalloproteinase-1 Promoter Polymorphisms with Prostate Cancer in Taiwanese Patients. Anticancer Res..

[16] Wu H.C., Chang C.H., Tsou Y.A., Tsai C.W., Lin C.C., Bau D.T. (2011). Significant association of caveolin-1 (CAV1) genotypes with prostate cancer susceptibility in Taiwan. Anticancer Res..

[17] Chang C.H., Chiu C.F., Wu H.C., Tseng H.C., Wang C.H., Lin C.C., Tsai C.W., Liang S.Y., Wang C.L., Bau D.T. (2008). Significant association of XRCC4 single nucleotide polymorphisms with prostate cancer susceptibility in Taiwanese males. Mol. Med. Rep..

[18] Mannino M.H., Zhu Z., Xiao H., Bai Q., Wakefield M.R., Fang Y. (2015). The paradoxical role of IL-10 in immunity and cancer. Cancer Lett..

[19] Ouyang W., O’Garra A. (2019). IL-10 Family Cytokines IL-10 and IL-22: From Basic Science to Clinical Translation. Immunity.

[20] Filella X., Alcover J., Zarco M.A., Beardo P., Molina R., Ballesta A.M. (2000). Analysis of type T1 and T2 cytokines in patients with prostate cancer. Prostate.

[21] Cardillo M.R., Ippoliti F. (2006). IL-6, IL-10 and HSP-90 expression in tissue microarrays from human prostate cancer assessed by computer-assisted image analysis. Anticancer Res..

[22] Dwivedi S., Goel A., Natu S.M., Mandhani A., Khattri S., Pant K.K. (2011). Diagnostic and prognostic significance of prostate specific antigen and serum interleukin 18 and 10 in patients with locally advanced prostate cancer: A prospective study. Asian Pac. J. Cancer Prev..

[23] Ugge H., Downer M.K., Carlsson J., Bowden M., Davidsson S., Mucci L.A., Fall K., Andersson S.O., Andren O. (2019). Circulating inflammation markers and prostate cancer. Prostate.

[24] Chen H., Zhou J., Luo J., Wu Y., Qian Y., Shi Y., Qu F., Shi B., Ding J., Cui X. (2022). Serum multi-cytokines screening identifies TRAIL and IL-10 as probable new biomarkers for prostate health index diagnostic utility adjustment in grey zone aggressive prostate cancer detection: A single-center data in China. Front. Immunol..

[25] Al-Nasralla A.S.H., Hussian S.S., Tektook N.K. (2023). Immunological analysis of Interleukin-10 (IL-10), tumor necrosis factor-a (TNF-a), and Prostate-specific antigen (PSA) in benign and malignant prostate cancer. Hum. Antibodies.

[26] Eskdale J., Kube D., Tesch H., Gallagher G. (1997). Mapping of the human IL10 gene and further characterization of the 5′ flanking sequence. Immunogenetics.

[27] Lowe P.R., Galley H.F., Abdel-Fattah A., Webster N.R. (2003). Influence of interleukin-10 polymorphisms on interleukin-10 expression and survival in critically ill patients. Crit. Care Med..

[28] Yao C.J., Du W., Chen H.B., Xiao S., Wang C.H., Fan Z.L. (2013). Associations of IL-10 gene polymorphisms with acute myeloid leukemia in Hunan, China. Asian Pac. J. Cancer Prev..

[29] Wang Y.C., He J.L., Tsai C.L., Tzeng H.E., Chang W.S., Pan S.H., Chen L.H., Su C.H., Lin J.C., Hung C.C. (2023). The Contribution of Tissue Inhibitor of Metalloproteinase-2 Genotypes to Breast Cancer Risk in Taiwan. Life.

[30] Chen C.H., Shih L.C., Hsu S.W., Tien H.C., Liu Y.F., Wang Y.C., Tsai C.W., Bau D.T., Chang W.S. (2024). Association of Matrix Metalloproteinase-9 Genotypes With Nasopharyngeal Carcinoma Risk. In Vivo.

[31] Chen K.Y., Chien W.C., Liao J.M., Tsai C.W., Chang W.S., Su C.H., Hsu S.W., Wang H.C., Bau D.T. (2021). Contribution of Interleukin-10 Genotype to Triple Negative Breast Cancer Risk. Anticancer Res..

[32] Gurel B., Lucia M.S., Thompson I.M., Goodman P.J., Tangen C.M., Kristal A.R., Parnes H.L., Hoque A., Lippman S.M., Sutcliffe S. (2014). Chronic inflammation in benign prostate tissue is associated with high-grade prostate cancer in the placebo arm of the prostate cancer prevention trial. Cancer Epidemiol. Biomark. Prev..

[33] Mason T.E., Ricks-Santi L., Chen W., Apprey V., Joykutty J., Ahaghotu C., Kittles R., Bonney G., Dunston G.M. (2010). Association of CD14 variant with prostate cancer in African American men. Prostate.

[34] De Marzo A.M., Nakai Y., Nelson W.G. (2007). Inflammation, atrophy, and prostate carcinogenesis. Urol. Oncol..

[35] De Marzo A.M., Platz E.A., Sutcliffe S., Xu J., Gronberg H., Drake C.G., Nakai Y., Isaacs W.B., Nelson W.G. (2007). Inflammation in prostate carcinogenesis. Nat. Rev. Cancer.

[36] Bakir W.A., Gaidan H.A., Al-Kaabi M.M. (2020). Immunohistochemical expression of interlukin10 (IL10) and heat shock protein-90 (HSP-90) in prostatic carcinoma. Indian J. Pathol. Microbiol..

[37] Gastl G.A., Abrams J.S., Nanus D.M., Oosterkamp R., Silver J., Liu F., Chen M., Albino A.P., Bander N.H. (1993). Interleukin-10 production by human carcinoma cell lines and its relationship to interleukin-6 expression. Int. J. Cancer.

[38] Verma S., Kushwaha P.P., Shankar E., Ponsky L.E., Gupta S. (2022). Increased cytokine gene expression and cognition risk associated with androgen deprivation therapy. Prostate.

[39] Hao N.B., Lu M.H., Fan Y.H., Cao Y.L., Zhang Z.R., Yang S.M. (2012). Macrophages in tumor microenvironments and the progression of tumors. Clin. Dev. Immunol..

[40] Moore K.W., de Waal Malefyt R., Coffman R.L., O’Garra A. (2001). Interleukin-10 and the interleukin-10 receptor. Annu. Rev. Immunol..

[41] Saraiva M., O’Garra A. (2010). The regulation of IL-10 production by immune cells. Nat. Rev. Immunol..

[42] Chu C.M., Chung C.J., Huang C.Y., Yu C.C., Wang C.H., Li L.F., Wu H.P. (2022). Serial Increases in Human Leukocyte Antigen-DR Expression and Decreases in Interleukin-10 Expression in Alveolar Monocytes of Survivors of Pneumonia-Related Acute Respiratory Distress Syndrome. Biology.

[43] Xu F., Yu S., Qin M., Mao Y., Jin L., Che N., Liu S., Ge R. (2018). Hydrogen-Rich Saline Ameliorates Allergic Rhinitis by Reversing the Imbalance of Th1/Th2 and Up-Regulation of CD4+CD25+Foxp3+Regulatory T Cells, Interleukin-10, and Membrane-Bound Transforming Growth Factor-beta in Guinea Pigs. Inflammation.

[44] Dennis K.L., Blatner N.R., Gounari F., Khazaie K. (2013). Current status of interleukin-10 and regulatory T-cells in cancer. Curr. Opin. Oncol..

[45] Stearns M.E., Hu Y., Wang M. (2003). IL-10 signaling via IL-10E1 is dependent on tyrosine phosphorylation in the IL-10R alpha chain in human primary prostate cancer cell lines. Oncogene.

[46] Chen Y., Song Y., Du W., Gong L., Chang H., Zou Z. (2019). Tumor-associated macrophages: An accomplice in solid tumor progression. J. Biomed. Sci..

[47] Bolpetti A., Silva J.S., Villa L.L., Lepique A.P. (2010). Interleukin-10 production by tumor infiltrating macrophages plays a role in Human Papillomavirus 16 tumor growth. BMC Immunol..

[48] Taube J.M., Young G.D., McMiller T.L., Chen S., Salas J.T., Pritchard T.S., Xu H., Meeker A.K., Fan J., Cheadle C. (2015). Differential Expression of Immune-Regulatory Genes Associated with PD-L1 Display in Melanoma: Implications for PD-1 Pathway Blockade. Clin. Cancer. Res..

[49] Butte M.J., Keir M.E., Phamduy T.B., Sharpe A.H., Freeman G.J. (2007). Programmed death-1 ligand 1 interacts specifically with the B7-1 costimulatory molecule to inhibit T cell responses. Immunity.

[50] Samiea A., Yoon J.S.J., Ong C.J., Zoubeidi A., Chamberlain T.C., Mui A.L. (2020). Interleukin-10 Induces Expression of Neuroendocrine Markers and PDL1 in Prostate Cancer Cells. Prostate Cancer.

[51] Horvat V., Mandic S., Marczi S., Mrcela M., Galic J. (2015). Association of IL-1beta and IL-10 Polymorphisms with Prostate Cancer Risk and Grade of Disease in Eastern Croatian Population. Coll. Antropol..

[52] Kesarwani P., Ahirwar D.K., Mandhani A., Singh A.N., Dalela D., Srivastava A.N., Mittal R.D. (2009). IL-10-1082 G>A: A risk for prostate cancer but may be protective against progression of prostate cancer in North Indian cohort. World J. Urol..

[53] Wang M.H., Helzlsouer K.J., Smith M.W., Hoffman-Bolton J.A., Clipp S.L., Grinberg V., De Marzo A.M., Isaacs W.B., Drake C.G., Shugart Y.Y. (2009). Association of IL10 and other immune response-and obesity-related genes with prostate cancer in CLUE II. Prostate.

[54] McCarron S.L., Edwards S., Evans P.R., Gibbs R., Dearnaley D.P., Dowe A., Southgate C., Easton D.F., Eeles R.A., Howell W.M. (2002). Influence of cytokine gene polymorphisms on the development of prostate cancer. Cancer Res..

[55] Bandil K., Singhal P., Dogra A., Rawal S.K., Doval D.C., Varshney A.K., Bharadwaj M. (2017). Association of SNPs/haplotypes in promoter of TNF A and IL-10 gene together with life style factors in prostate cancer progression in Indian population. Inflamm. Res..

[56] Winchester D.A., Till C., Goodman P.J., Tangen C.M., Santella R.M., Johnson-Pais T.L., Leach R.J., Xu J., Zheng S.L., Thompson I.M. (2017). Association between variants in genes involved in the immune response and prostate cancer risk in men randomized to the finasteride arm in the Prostate Cancer Prevention Trial. Prostate.

[57] Dluzniewski P.J., Wang M.H., Zheng S.L., De Marzo A.M., Drake C.G., Fedor H.L., Partin A.W., Han M., Fallin M.D., Xu J. (2012). Variation in IL10 and other genes involved in the immune response and in oxidation and prostate cancer recurrence. Cancer Epidemiol. Biomark. Prev..

[58] VanCleave T.T., Moore J.H., Benford M.L., Brock G.N., Kalbfleisch T., Baumgartner R.N., Lillard J.W., Kittles R.A., Kidd L.C. (2010). Interaction among variant vascular endothelial growth factor (VEGF) and its receptor in relation to prostate cancer risk. Prostate.

[59] Zabaleta J., Lin H.Y., Sierra R.A., Hall M.C., Clark P.E., Sartor O.A., Hu J.J., Ochoa A.C. (2008). Interactions of cytokine gene polymorphisms in prostate cancer risk. Carcinogenesis.

[60] Faupel-Badger J.M., Kidd L.C., Albanes D., Virtamo J., Woodson K., Tangrea J.A. (2008). Association of IL-10 polymorphisms with prostate cancer risk and grade of disease. Cancer Causes Control.

[61] Michaud D.S., Daugherty S.E., Berndt S.I., Platz E.A., Yeager M., Crawford E.D., Hsing A., Huang W.Y., Hayes R.B. (2006). Genetic polymorphisms of interleukin-1B (IL-1B), IL-6, IL-8, and IL-10 and risk of prostate cancer. Cancer Res..

[62] Xu J., Lowey J., Wiklund F., Sun J., Lindmark F., Hsu F.C., Dimitrov L., Chang B., Turner A.R., Liu W. (2005). The interaction of four genes in the inflammation pathway significantly predicts prostate cancer risk. Cancer Epidemiol. Biomark. Prev..

[63] Liu J., Song B., Bai X., Liu W., Li Z., Wang J., Zheng Y., Wang Z. (2010). Association of genetic polymorphisms in the interleukin-10 promoter with risk of prostate cancer in Chinese. BMC Cancer.

[64] Lv C., Wang Y., Wang J., Zhang H., Xu H., Zhang D. (2011). Association of Interleukin-10 gene polymorphisms with ankylosing spondylitis. Clin. Investig. Med..

[65] Miteva L.D., Stanilov N.S., Deliysky T.S., Stanilova S.A. (2014). Significance of -1082A/G polymorphism of IL10 gene for progression of colorectal cancer and IL-10 expression. Tumor Biol..

[66] Fathy M.M., Elsaadany H.F., Ali Y.F., Farghaly M.A., Hamed M.E., Ibrahim H.E., Noah M.A., Allah M.A., Elashkar S.S., Abdelsalam N.I. (2017). Association of IL-10 gene polymorphisms and susceptibility to Juvenile Idiopathic Arthritis in Egyptian children and adolescents: A case-control study. Ital. J. Pediatr..

[67] Azab S.F., Abdalhady M.A., Elsaadany H.F., Elkomi M.A., Elhindawy E.M., Sarhan D.T., Salam M.M., Allah M.A., Emam A.A., Noah M.A. (2016). Interleukin-10-1082 G/A gene polymorphisms in Egyptian children with CAP: A case-control study. Medicine.

[68] Reuss E., Fimmers R., Kruger A., Becker C., Rittner C., Hohler T. (2002). Differential regulation of interleukin-10 production by genetic and environmental factors--a twin study. Genes Immun..

[69] Larsson L., Johansson P., Jansson A., Donati M., Rymo L., Berglundh T. (2009). The Sp1 transcription factor binds to the G-allele of the-1087 IL-10 gene polymorphism and enhances transcriptional activation. Genes Immun..

[70] Larsson L., Rymo L., Berglundh T. (2010). Sp1 binds to the G allele of the-1087 polymorphism in the IL-10 promoter and promotes IL-10 mRNA transcription and protein production. Genes Immun..

[71] Larsson L., Thorbert-Mros S., Rymo L., Berglundh T. (2011). Interleukin-10 genotypes of the -1087 single nucleotide polymorphism influence sp1 expression in periodontitis lesions. J. Periodontol..

[72] Borras F.E., Lloberas J., Maki R.A., Celada A. (1995). Repression of I-A beta gene expression by the transcription factor PU.1. J. Biol. Chem..

[73] Harendza S., Lovett D.H., Stahl R.A. (2000). The hematopoietic transcription factor PU.1 represses gelatinase A transcription in glomerular mesangial cells. J. Biol. Chem..

[74] Dwivedi S., Goel A., Khattri S., Mandhani A., Sharma P., Misra S., Pant K.K. (2015). Genetic variability at promoters of IL-18 (pro-) and IL-10 (anti-) inflammatory gene affects susceptibility and their circulating serum levels: An explorative study of prostate cancer patients in North Indian populations. Cytokine.

[75] Men T., Yu C., Wang D., Liu F., Li J., Qi X., Yang C., Jiang W., Wei X., Li X. (2017). The impact of interleukin-10 (IL-10) gene 4 polymorphisms on peripheral blood IL-10 variation and prostate cancer risk based on published studies. Oncotarget.

[76] Tsai C.W., Chang W.S., Lin K.C., Shih L.C., Tsai M.H., Hsiao C.L., Yang M.D., Lin C.C., Bau D.T. (2014). Significant association of Interleukin-10 genotypes and oral cancer susceptibility in Taiwan. Anticancer Res..

[77] You Y., Du X., Fan M., Wu B., Xie C. (2015). Association between IL-10 polymorphisms (-1082A/G, -592A/C and -819T/C) and oral cancer risk. Int. J. Clin. Exp. Med..

[78] Tsai C.W., Tsai M.H., Shih L.C., Chang W.S., Lin C.C., Bau D.T. (2013). Association of interleukin-10 (IL10) promoter genotypes with nasopharyngeal carcinoma risk in Taiwan. Anticancer Res..

[79] Cil E., Kumral A., Kanmaz-Ozer M., Vural P., Dogru-Abbasoglu S., Altuntas Y., Uysal M. (2014). Interleukin-10-1082 gene polymorphism is associated with papillary thyroid cancer. Mol. Biol. Rep..

[80] Erdogan M., Karadeniz M., Ozbek M., Ozgen A.G., Berdeli A. (2008). Interleukin-10 gene polymorphism in patients with papillary thyroid cancer in Turkish population. J. Endocrinol. Investig..

[81] Sun F., Sun Y., Zhang D., Zhang J., Song B., Zheng H. (2010). Association of interleukin-10 gene polymorphism with cachexia in Chinese patients with gastric cancer. Ann. Clin. Lab. Sci..

[82] Kuo W.H., Huang C.Y., Fu C.K., Hsieh Y.H., Liao C.H., Hsu C.M., Huang Y.K., Tsai C.W., Chang W.S., Bau D.T. (2014). Effects of interleukin-10 polymorphisms and smoking on the risk of gastric cancer in Taiwan. In Vivo.

[83] Cardenas D.M., Sanchez A.C., Rosas D.A., Rivero E., Paparoni M.D., Cruz M.A., Suarez Y.P., Galvis N.F. (2018). Preliminary analysis of single-nucleotide polymorphisms in IL-10, IL-4, and IL-4Ralpha genes and profile of circulating cytokines in patients with gastric Cancer. BMC Gastroenterol..

[84] Shih C.M., Lee Y.L., Chiou H.L., Hsu W.F., Chen W.E., Chou M.C., Lin L.Y. (2005). The involvement of genetic polymorphism of IL-10 promoter in non-small cell lung cancer. Lung Cancer.

[85] Pooja S., Chaudhary P., Nayak L.V., Rajender S., Saini K.S., Deol D., Kumar S., Bid H.K., Konwar R. (2012). Polymorphic variations in IL-1beta, IL-6 and IL-10 genes, their circulating serum levels and breast cancer risk in Indian women. Cytokine.

[86] Havranek E., Howell W.M., Fussell H.M., Whelan J.A., Whelan M.A., Pandha H.S. (2005). An interleukin-10 promoter polymorphism may influence tumor development in renal cell carcinoma. J. Urol..

[87] Romero J.M., Saenz-Lopez P., Cozar J.M., Carretero R., Canton J., Vazquez F., Concha A., Tallada M., Garrido F., Ruiz-Cabello F. (2009). A polymorphism in the interleukin-10 promoter affects the course of disease in patients with clear-cell renal carcinoma. Hum. Immunol..

[88] Khorrami S., Zamani H., Hasanzadeh M., Mehramiz M., Soleimani A., Zare Marzouni H., Ferns G.A., Esmaeili H., Avan A. (2022). Association of a genetic variant in Interleukin-10 gene with increased risk and inflammation associated with cervical cancer. Gene.

[89] Pratap P.D., Raza S.T., Zaidi G., Kunwar S., Ahmad S., Charles M.R., Eba A., Rajput M. (2022). Genetic Variants in Interleukin-10 Gene Association with Susceptibility and Cervical Cancer Development: A Case Control Study. Glob. Med. Genet..

[90] Nedoszytko B., Olszewska B., Roszkiewicz J., Glen J., Zablotna M., Lugowska-Umer H., Nowicki R., Sokolowska-Wojdylo M. (2016). The role of polymorphism of interleukin-2, -10, -13 and TNF-alpha genes in cutaneous T-cell lymphoma pathogenesis. Adv. Dermatol. Allergol./Postępy Dermatol. Alergol..

[91] Dai Z.M., He A.L., Zhang W.G., Liu J., Cao X.M., Chen Y.X., Ma X.R., Zhao W.H., Dai Z.J. (2014). Association of the four common polymorphisms in interleukin-10 (rs1800890, rs1800896, rs1800871, and rs1800872) with non-Hodgkin’s lymphoma risk: A meta-analysis. Int. J. Clin. Exp. Med..

[92] Ovsepyan V.A., Gabdulkhakova A., Shubenkiva A.A., Zotina E.N. (2015). Role of Interleukin-10 Gene Promoter Region Polymorphism in the Development of Chronic Lymphoid Leukemia. Bull. Exp. Biol. Med..

[93] El Baiomy M.A., Akl T., ElMenshawy N., El-Sebaie A.H., Morkos H., El-Ashwah S., El-Sabbagh A.M., Wahba Y., El-Ghonemy M. (2023). The prognostic values of the IL-10 (G1082A) and TNF-alpha (G308A) polymorphisms in Egyptian patients with acute lymphoblastic leukemia: A single-center study. Indian J. Cancer.

[94] Martinez-Campos C., Torres-Poveda K., Camorlinga-Ponce M., Flores-Luna L., Maldonado-Bernal C., Madrid-Marina V., Torres J. (2019). Polymorphisms in IL-10 and TGF-beta gene promoter are associated with lower risk to gastric cancer in a Mexican population. BMC Cancer.

[95] Xue H., Lin B., An J., Zhu Y., Huang G. (2012). Interleukin-10-819 promoter polymorphism in association with gastric cancer risk. BMC Cancer.

[96] Chang W.S., Liao C.H., Tsai C.W., Hu P.S., Wu H.C., Hsu S.W., Ji H.X., Hsiao C.L., Bau D.T. (2016). The Role of IL-10 Promoter Polymorphisms in Renal Cell Carcinoma. Anticancer Res..

[97] Hsia T.C., Chang W.S., Liang S.J., Chen W.C., Tu C.Y., Chen H.J., Yang M.D., Tsai C.W., Hsu C.M., Tsai C.H. (2014). Interleukin-10 (IL-10) promoter genotypes are associated with lung cancer risk in Taiwan males and smokers. Anticancer Res..

[98] Bai C.Y., Shi X.Y., He J., Xue J., Feng Y. (2016). Association between IL-10 genetic variations and cervical cancer susceptibility in a Chinese population. Genet. Mol. Res..

[99] Ahirwar D., Mandhani A., Mittal R.D. (2009). Interleukin-10 G-1082A and C-819T polymorphisms as possible molecular markers of urothelial bladder cancer. Arch. Med. Res..

[100] Fei C., Yao X.M., Sun Y., Gu X.Z., Yu L.Q., Lai X. (2015). Interleukin-10 polymorphisms associated with susceptibility to acute myeloid leukemia. Genet. Mol. Res..

[101] Singh P.K., Ahmad M.K., Kumar V., Gupta R., Kohli M., Jain A., Mahdi A.A., Bogra J., Chandra G. (2017). Genetic polymorphism of interleukin-10 (-A592C) among oral cancer with squamous cell carcinoma. Arch. Oral. Biol..

[102] Sun J.M., Li Q., Gu H.Y., Chen Y.J., Wei J.S., Zhu Q., Chen L. (2013). Interleukin 10 rs1800872 T>G polymorphism was associated with an increased risk of esophageal cancer in a Chinese population. Asian Pac. J. Cancer Prev..

[103] Qi M., Liu D.M., Pan L.L., Lin Y.X. (2014). Interleukin-10 gene -592C>A polymorphism and susceptibility to gastric cancer. Genet. Mol. Res..

[104] Gallegos-Arreola M.P., Zuniga Gonzalez G.M., Figuera L.E., Puebla-Perez A.M., Delgado Saucedo J.I. (2019). Association of the IL-10 gene rs1800872 (-592 C>A) polymorphism with breast cancer in a Mexican population. JBUON.

[105] Du G.H., Wang J.K., Richards J.R., Wang J.J. (2019). Genetic polymorphisms in tumor necrosis factor alpha and interleukin-10 are associated with an increased risk of cervical cancer. Int. Immunopharmacol..

[106] Duvlis S., Dabeski D., Noveski P., Ivkovski L., Plaseska-Karanfilska D. (2020). Association of IL-10 (rs1800872) and IL-4R (rs1805010) polymorphisms with cervical intraepithelial lesions and cervical carcinomas. JBUON.

[107] Gaiolla R.D., Moraes M.P.T., de Oliveira D.E. (2021). SNPs in genes encoding for IL-10, TNF-alpha, and NFkappaB p105/p50 are associated with clinical prognostic factors for patients with Hodgkin lymphoma. PLoS ONE.

[108] Saraiva M., Vieira P., O’Garra A. (2020). Biology and therapeutic potential of interleukin-10. J. Exp. Med..

[109] Wang X., Wong K., Ouyang W., Rutz S. (2019). Targeting IL-10 Family Cytokines for the Treatment of Human Diseases. Cold Spring Harb. Perspect. Biol..

[110] Hagenbaugh A., Sharma S., Dubinett S.M., Wei S.H., Aranda R., Cheroutre H., Fowell D.J., Binder S., Tsao B., Locksley R.M. (1997). Altered immune responses in interleukin 10 transgenic mice. J. Exp. Med..

[111] Loser K., Apelt J., Voskort M., Mohaupt M., Balkow S., Schwarz T., Grabbe S., Beissert S. (2007). IL-10 controls ultraviolet-induced carcinogenesis in mice. J. Immunol..

[112] Ruffell B., Chang-Strachan D., Chan V., Rosenbusch A., Ho C.M., Pryer N., Daniel D., Hwang E.S., Rugo H.S., Coussens L.M. (2014). Macrophage IL-10 blocks CD8+ T cell-dependent responses to chemotherapy by suppressing IL-12 expression in intratumoral dendritic cells. Cancer Cell.

[113] Sullivan K.M., Jiang X., Guha P., Lausted C., Carter J.A., Hsu C., Labadie K.P., Kohli K., Kenerson H.L., Daniel S.K. (2023). Blockade of interleukin 10 potentiates antitumour immune function in human colorectal cancer liver metastases. Gut.

[114] Labadie K.P., Kreuser S.A., Brempelis K.J., Daniel S.K., Jiang X., Sullivan K.M., Utria A.F., Kenerson H.L., Kim T.S., Crane C.A. (2023). Production of an interleukin-10 blocking antibody by genetically engineered macrophages increases cancer cell death in human gastrointestinal tumor slice cultures. Cancer Gene Ther..

[115] Naing A., Papadopoulos K.P., Autio K.A., Ott P.A., Patel M.R., Wong D.J., Falchook G.S., Pant S., Whiteside M., Rasco D.R. (2016). Safety, Antitumor Activity, and Immune Activation of Pegylated Recombinant Human Interleukin-10 (AM0010) in Patients With Advanced Solid Tumors. J. Clin. Oncol..

[116] Porcaro A.B., Bianchi A., Gallina S., Panunzio A., Serafin E., Mazzucato G., Orlando R., Montanaro F., Patuzzo G.M., Baielli A. (2023). Advanced age is an independent prognostic factor of disease progression in high-risk prostate cancer: Results in 180 patients treated with robot-assisted radical prostatectomy and extended pelvic lymph node dissection in a tertiary referral center. Aging. Clin. Exp. Res..

[117] Defever K., Platz E.A., Lopez D.S., Mondul A.M. (2020). Differences in the prevalence of modifiable risk and protective factors for prostate cancer by race and ethnicity in the National Health and Nutrition Examination Survey. Cancer Causes Control.

[118] Stone S.N., Hoffman R.M., Tollestrup K., Stidley C.A., Witter J.L., Gilliland F.D. (2003). Family history, Hispanic ethnicity, and prostate cancer risk. Ethn. Dis..

[119] Huang J., Sun J., Wang K., Zheng L., Fan Y., Qian B. (2024). Causal relationship between prostatic diseases and prostate cancer: A mendelian randomization study. BMC Cancer.

[120] Di Maso M., Augustin L.S.A., Jenkins D.J.A., Carioli G., Turati F., Grisoni B., Crispo A., La Vecchia C., Serraino D., Polesel J. (2022). Adherence to a cholesterol-lowering diet and the risk of prostate cancer. Food Funct..

[121] Ellis E.T., Fairman B.J., Stahr S.D., Bensen J.T., Mohler J.L., Song L., Butler E.N., Su L.J., Hsu P.C. (2024). Cigarette smoking and prostate cancer aggressiveness among African and European American men. Cancer Causes Control.

[122] Brookman-May S.D., Campi R., Henriquez J.D.S., Klatte T., Langenhuijsen J.F., Brausi M., Linares-Espinos E., Volpe A., Marszalek M., Akdogan B. (2019). Latest Evidence on the Impact of Smoking, Sports, and Sexual Activity as Modifiable Lifestyle Risk Factors for Prostate Cancer Incidence, Recurrence, and Progression: A Systematic Review of the Literature by the European Association of Urology Section of Oncological Urology (ESOU). Eur. Urol. Focus.

[123] Al-Fayez S., El-Metwally A. (2023). Cigarette smoking and prostate cancer: A systematic review and meta-analysis of prospective cohort studies. Tob. Induc. Dis..

[124] Cui H., Zhang W., Zhang L., Qu Y., Xu Z., Tan Z., Yan P., Tang M., Yang C., Wang Y. (2024). Risk factors for prostate cancer: An umbrella review of prospective observational studies and mendelian randomization analyses. PLoS Med..

